# Estimation of hemoglobin concentration at the initiation of cardiopulmonary bypass using support vector regression

**DOI:** 10.1051/ject/2025071

**Published:** 2026-06-19

**Authors:** Harutoyo Hirano, Shun Takahashi, Tetsuya Kamei, Makoto Hibiya

**Affiliations:** 1 Department of Medical Equipment Engineering, Clinical and Educational Collaboration Unit, Faculty of Medical Sciences, Fujita Health University Toyoake Aichi Japan; 2 Department of Biomedical Engineering, Graduate School of Medical Science, Fujita Health University Toyoake Aichi Japan; 3 Fundamental Education Department, Faculty of Medical Sciences, Fujita Health University Toyoake Aichi Japan; 4 Department of Clinical Engineering, Clinical and Educational Collaboration Unit, Faculty of Medical Sciences, Fujita Health University Toyoake Aichi Japan

**Keywords:** Hemoglobin, Support vector regression, Cardiopulmonary bypass, Machine learning, Hemodilution

## Abstract

*Background*: Hemodilution during cardiopulmonary bypass (CPB) is a standard perfusion strategy used to reduce blood viscosity and enhance microcirculatory flow. The hemodilution rate, expressed as hemoglobin (Hb) concentration, is a key control index in CPB and is currently estimated from total blood volume (TBV). The objective of this study was to propose a novel formula to accurately predict Hb concentration at the initiation of CPB (Hb_CPB_) by incorporating circulating blood volume, laboratory data, physical measurements, and patient history. *Methods*: We retrospectively analyzed 577 adult patients who underwent elective CPB at Fujita Health University Hospital from January 2016 to December 2020. Thirty-six preoperative variables – including demographics, laboratory data, circuit parameters, and indices such as TBV and ideal weight – were standardized. Categorical variables underwent one-hot encoding. We compared generalized linear models (GLM), support vector regression (SVR), and multilayer perceptron (MLP). Model performance was evaluated using the coefficient of determination (*R*^2^), mean squared error (MSE), and Bland–Altman analysis (bias and 95% limits of agreement [LoA]). Predictions from two conventional TBV-based formulas were used as benchmarks. *Results*: Of 993 screened cases, 577 met inclusion criteria (447 males, mean age 66.8 ± 11.7 years; 130 females, 69.5 ± 10.6 years). SVR on standardized predictors achieved the highest accuracy (*R*^2^ = 0.498, MSE = 0.517), outperforming GLM (*R*^2^ = 0.429, MSE = 0.797) and MLP (*R*^2^ = 0.332, MSE = 0.669). Conventional formulas showed lower performance (*R*^2^ = 0.325, MSE ≥ 1.48). Bland–Altman analysis for SVR demonstrated minimal bias (–0.0028 g/dL) and narrower LoA (–1.42 to 1.41 g/dL) than conventional methods (bias –1.33 g/dL; LoA –3.49 to 0.83 g/dL). *Conclusion*: These findings suggest that an SVR-based model improves prediction of Hb_CPB_ over conventional approaches, supporting optimized transfusion management and reduced hemodilution-related risks.

## Introduction

Japan’s population is aging rapidly, and the proportion of elderly patients undergoing cardiac surgery with cardiopulmonary bypass (CPB) has steadily increased over the past decade [1–4]. As this trend is expected to continue, developing appropriate CPB technologies for older patients has become imperative. Hemodilution during CPB is a standard perfusion strategy used to reduce blood viscosity and enhance microcirculatory flow [5]. However, anemia resulting from hemodilution may cause ischemic organ injury, increase mortality, and elevate stroke risk [6–8]. Current guidelines recommend maintaining hematocrit (Ht) levels ≥ 21% and/or hemoglobin (Hb) concentrations ≥ 7.0 g/dL during CPB to minimize hemodilution-related complications [9–11]. To assess the hemodilution rate, total blood volume (TBV) is estimated using body weight in kilograms (kg). TBV estimates are typically set at 70 mL/kg for American adult males, 65 mL/kg for females [12, 13], and 80 mL/kg for Japanese adults [14]. This method is commonly applied to all adults, regardless of age. However, several studies have shown discrepancies between predicted post-dilution Ht or Hb values and actual Hb concentrations at the initiation of CPB (Hb_CPB_) [15–17]. Revised formulas incorporating sex and age have been proposed to improve TBV estimates [18]. Nonetheless, these methods often lack applicability to older patients, as they do not adequately account for age-related TBV decline [19].

This study aims to develop and propose a novel formula to predict ${\mathrm{Hb}}_{\mathrm{CPB}}$ more accurately by incorporating TBV, laboratory data, physical measurements, and clinical history.

## Materials and methods

### Clinical data

**Figure 1:**
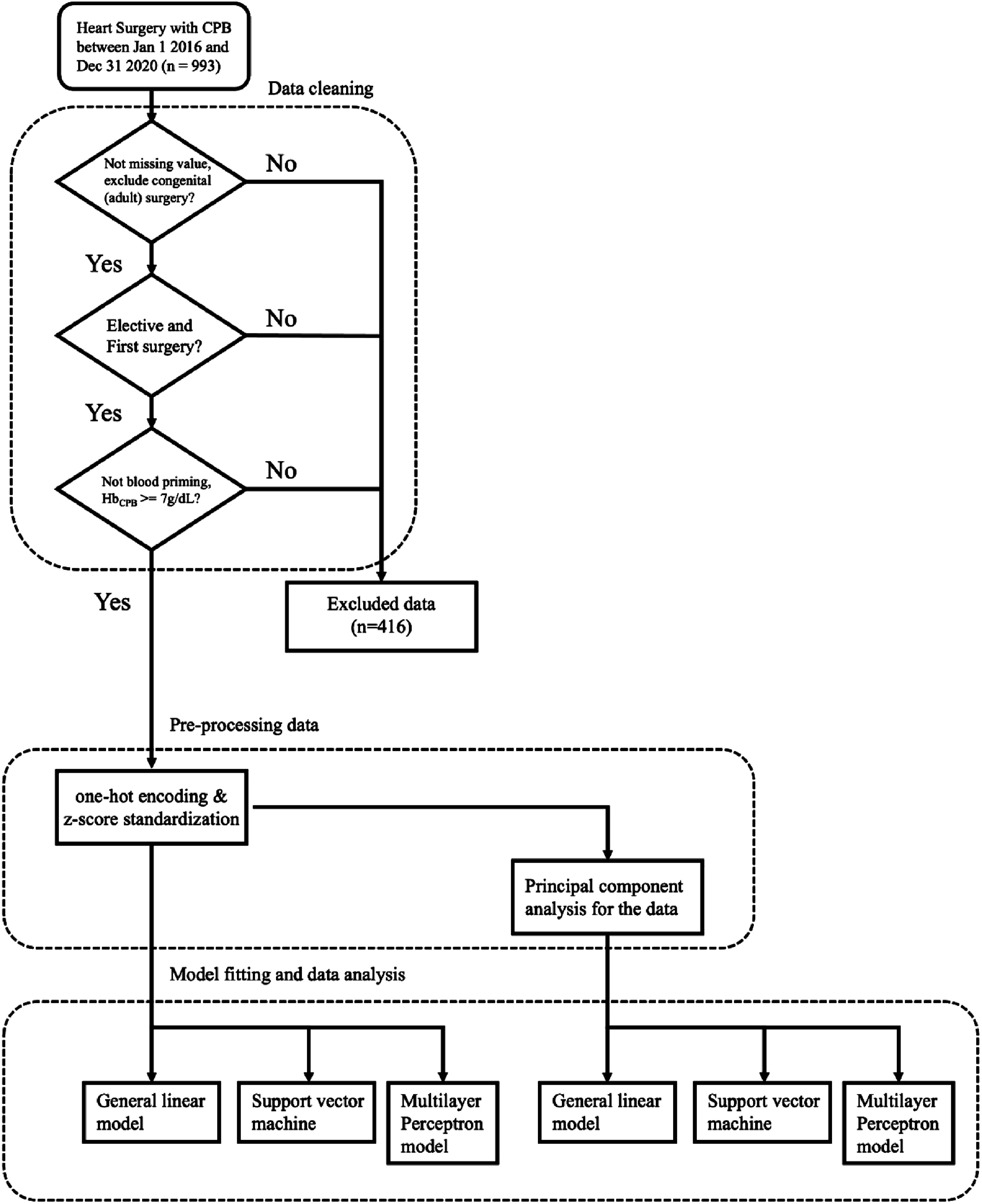
Analysis flowchart.

This study analyzed data that were prepared to register in the Perfusion Database maintained by the Japanese Society of Extra-Corporeal Technology in Medicine (JaSECT) for patients who underwent cardiac surgery with CPB at Fujita Health University Hospital between January 1, 2016, and December 31, 2020. Only elective cases were included. Patients with congenital heart disease or those who underwent repeat surgeries were excluded to avoid duplicate entries, particularly in preoperative background data. Additionally, cases involving blood priming or with hemoglobin (Hb) concentrations below 7 g/dL at CPB initiation were excluded. Details of the data cleaning process are shown in Figure 1. The JaSECT Perfusion Database collects data across six domains: patient demographics; circuit and priming fluid details; CPB parameters; fluid volume input and output management; laboratory data and outcomes. These domains consist of 84 multiple-choice items and 159 numeric variables, totaling 243 parameters [20]. From in-hospital data for this database, we extracted 35 pre-CPB parameters along with Hb_CPB_ for model development, as described in the following section.

- *Demographic and clinical variables*: sex; age at surgery; surgical procedure; height; weight; left ventricular ejection fraction; and risk factors, including congestive heart failure, chronic respiratory disease, smoking history, diabetes mellitus, arrhythmia, hypertension, dyslipidemia, cardiovascular or extracardiac vascular disease, cerebrovascular disease, renal dysfunction, and chronic dialysis.
- *Blood laboratory data obtained by the time of admission to the operating room*: glucose (mg/dL); potassium (mEq/L); lactate (mg/dL); creatinine (mg/dL); Hb (g/dL); results of arterial blood gas analysis (pH, PaO_2_(mmHg), PaCO_2_(mmHg), HCO_3_^−^ (mEq/L)) and activated clotting time (sec)
- *Variables described the CPB circuit configuration*: biocompatible coating part; planned priming volume (Static circuit volume); and priming fluid composition.
- *Variables obtained from formulas using collected data*: ideal body weight; TBV; body mass index (BMI); obesity classification; and body surface area (BSA).

BMI, ideal body weight (BW_i_) [18], TBV [11] and BSA [21] were calculated as follows (Eqs. (1)–(4)):

(1) $$\mathrm{BMI}\;=\frac{w}{h^{2}},$$

(2) $${\mathrm{BW}}_{i}=22h^{2},$$

(3) TBV=80w,

(4) $$\mathrm{BSA}=0.7184\times 10^{-4}\times h^{0.725}\times w^{0.425},$$

where *w* is the body weight (kg) and *h* is the height (m).

Obesity was classified according to the Japanese Society for the Study of Obesity Guidelines [22] as follows: underweight (BMI < 18.5 kg/m^2^), normal weight (18.5 ≤ BMI < 25 kg/m^2^), obesity class I (25 ≤ BMI < 30 kg/m^2^), class II (30 ≤ BMI < 35 kg/m^2^), class III (35 ≤ BMI < 40 kg/m^2^), and class IV (BMI ≥ 40 kg/m^2^). Hematocrit (Ht) values measured before and at CPB initiation were converted to Hb concentrations using the equation: Ht ≈ 3 × Hb [23].

### Analysis protocol

#### Preprocessing of eligible clinical data

The 35 parameters described in the previous section were considered candidate explanatory variables for estimating Hb_CPB_. One-hot encoding was applied to 15 categorical variables (e.g., sex, type of surgery, and presence of comorbidities), and z-score standardization was applied to 20 continuous variables (e.g., laboratory data) [24, 25]. The interactions among variables, and between each variable and hemoglobin concentration, remain unknown. In general, including variables with low or no relevance to the target variable may impair the generalizability of a predictive model and lead to overfitting. It is therefore advisable to limit explanatory variables to those that carry significant information about the outcome [26]. However, this restriction may exclude potentially informative features. To address this, we prepared two datasets for analysis: the full standardized dataset and a dimensionally reduced dataset, described below.

Principal Component Analysis (PCA) [27] was applied to the standardized explanatory matrix **X**∈R^*N*×*P*^ (where *N* is the number of subjects and *P* is the number of variables) to mitigate multicollinearity. Eigenvalue decomposition was performed on the covariance matrix $\Sigma =\frac{1}{N-1},\;X^{T}X$, and the smallest number of principal components *K* was selected such that the cumulative contribution of the eigenvalues ${\lambda_{k}}_{k=1}^{P}$ exceeded 90%. Next, the principal component score matrix $P=\left[t_{1},\ldots ,t_{K},\right]=\;X\left[v_{1},\ldots ,v_{K}\right]\in R^{N\times K}$was calculated using the principal component vector ${v_{k}}_{k=1}^{K}$. After PCA, **P** was used as the explanatory variable for the subsequent model training.

#### Linear model for predicting hemoglobin concentration at CPB initiation

Conventional methods for estimating Hb_CPB_ [17–19] are essentially linear models that incorporate a limited set of patient and circuit factors, such as priming volume. We first evaluated whether a linear approach would be adequate for our dataset. To do so, we fit a generalized linear model (GLM) using routinely available pre-CPB variables, including body size indices, laboratory values, and circuit settings. The GLM assumes that Hb_CPB_ varies in approximate proportion to changes in these inputs. Model performance was assessed using the coefficient of determination (*R*^2^) and mean squared error (MSE). The explicit GLM equation and estimation procedures are provided in Supplementary equation (s-i).

#### Nonlinear model for predicting hemoglobin concentration at CPB initiation

Because clinical data often contain nonlinear relationships and interactions that linear models may not capture, we also evaluated two standard nonlinear approaches: Support Vector Regression (SVR) and a Multilayer Perceptron (MLP).

SVR can model curved relationships between the inputs and Hb_CPB_ without the need to specify functional forms. We used a widely adopted SVR implementation and tuned its hyperparameters by cross-validation to reduce overfitting; further details are provided in the Supplementary equations S-ii, iii). The MLP is a simple neural-network model that learns patterns by stacking a small number of layers. We compared several compact architectures and training configurations and selected the model that performed best in cross-validation. Training details (architecture and stopping criteria) are summarized in the Supplementary equation S-iv [28–30].

For some analyses, we also used a compressed set of input variables derived from principal component analysis to reduce redundancy among predictors. We retained the smallest number of components that preserved approximately 90% of the total variance. Technical details are provided in Supplementary material S2.

#### Model training, validation, and reporting

For all models, we standardized inputs when appropriate, tuned model settings by cross-validation, and summarized performance using *R*^2^ and MSE. Because correlation-based indices do not measure exact agreement, we also evaluated agreement between predicted and measured Hb_CPB_ with Bland–Altman analysis (bias and 95% limits of agreement) (Supplementary material S5).

#### Software and reproducibility

Analyses were performed with commonly used, off-the-shelf software (e.g., MATLAB equivalents or open-source alternatives). Parameter settings and code snippets sufficient for reproduction are listed in the Supplementary material.

#### Evaluation of the proposed method

To evaluate the performance of the trained models, predictions on the validation and hold-out test sets were computed and compared against the reference values of Hb_CPB_. As this is a regression task, three metrics were used to quantify prediction accuracy: MSE, mean absolute error, and the coefficient of determination (*R*^2^). In addition, Bland–Altman analysis was performed to further examine the agreement between predicted and measured hemoglobin concentrations. From this analysis, the mean difference (bias) and 95% limits of agreement (LoA), defined as ±1.96 times the standard deviation of the differences, were calculated.

To benchmark our method against conventional prediction models, the same clinical dataset was fitted using the standard hematocrit-based prediction formula [18], which is expressed as:

(5) $$Ht_{\mathrm{CPB}}={\mathrm{Ht}}_{\mathrm{beforeCPB}}\times \left(1-\frac{\mathrm{PV}}{\mathrm{PV}+\mathrm{TBV}}\right),$$

where Ht_CPB_ is the hematocrit value under CPB, Ht_beforeCPB_ is the hematocrit value before CPB, PV is Priming Volume. TBV was substituted from two approaches: equation (3) [19] and the following equation [18]:

(6) TBV=70×BW (Age<65)60×BW (Age≥65),

Estimated Hb concentrations based on equation (5) and their Bland–Altman statistics, mean difference, and 95% LoA, were compared with those obtained for our approach.

## Results

Data from 577 patients, including 447 males (mean age 66.1 ± 11.8 years) and 130 females (mean age 69.5 ± 10.6 years), were included in the analysis. Subject characteristics are summarized in Table 1. In terms of obesity classification, 31 patients (5.4%) were underweight, 361 (62.6%) were standard weight, 146 (25.3%) were classified as obese class I, 33 (5.7%) as class II, 5 (0.8%) as class III, and 1 (0.2%) as class IV. The surgical procedures included coronary artery bypass grafting (CABG) in 149 patients (25.8%), valve surgery in 215 (37.3%), combined CABG and valve procedures in 67 (11.6%), combined CABG and other procedures in 27 (4.7%), aortic surgery in 91 (15.8%), and other procedures in 28 (4.8%). Regarding biopassive coating, it was absent in 2 patients (0.3%), applied to limited components in 34 (5.9%), applied to all components except the cannulae in 484 (84.0%), and applied tip-to-tip in 57 (9.8%). The main priming solution was bicarbonate Ringer’s in 540 patients (93.6%). Other solutions included saline in 17 (3.0%), lactated Ringer’s in 2 (0.3%), acetate Ringer’s in 3 (0.5%), starch or dextran in 3 (0.5%), and other crystalloid-only solutions in 12 (2.1%). A centrifugal pump was employed in all cases.

**Table 1:** Clinical characteristics/Baseline clinical characteristics of the study population.

Index	Total (*n* = 577)	Male (*n* = 447)	Female (*n* = 130)
Demographics			
Age of surgery (years)	66.9 ± 11.6	66.1 ± 11.8	69.5 ± 10.6
Height (cm)	163.0 ± 9.2	167.0 ± 7.31	153.0 ± 6.42
Weight (kg)	63.6 ± 12.3	66.2 ± 11.5	54.8 ± 10.5
Body mass index (kg/m^2^)	23.8 ± 3.8	23.8 ± 3.66	23.5 ± 4.16
Body surface area (m^2^)	1.68 ± 0.18	1.74 ± 0.16	1.50 ± 0.14
Activated clotting time (s)	117.0 ± 17.3	118.0 ± 17.0	114.0 ± 18.0

LV fraction			
Good	202 (35.0%)	140 (31.3%)	62 (47.7%)
Med	336 (58.2%)	275 (61.5%)	61 (46.9%)
Bad	39 (6.8%)	32 (7.2%)	7 (5.4%)

Laboratory data			
Creatinine (mg/dL)	0.97 [0.47]	1.01 [0.44]	0.76 [0.39]
Glucose (mg/dL)	109 [26]	109 [26]	110 [23]
Potassium (mEq/L)	3.9 [0.5]	3.9 [0.6]	3.8 [0.6]
Lactic acid (mg/dL)	5.5 [3.4]	5.6 [3.3]	5.0 [3.6]
Hb (g/dL, before CPB)	12.6 [1.8]	12.7 [1.85]	11.9 [1.6]
Hb (g/dL, initiation of CPB)	8.3 [1.4]	8.5 [1.4]	7.8 [1.1]
pH	7.39 [0.07]	7.39 [0.07]	7.40 [0.07]
Po_2_ (mmHg)	450 [219]	456 [214]	419 [263]
Pco_2_ (mmHg)	44.2 [9.30]	44.9 [9.40]	42.3 [8.15]
HCO_3_^−^ (mEq/L)	26.8 [3.30]	26.8 [3.30]	26.8 [3.75]

Blood volume estimations			
Ideal weight (kg)	58*.*9 ± 6*.*6	61*.*1 ± 5*.*4	51*.*3 ± 4*.*3
TBV (mL)	5088 ± 981	5293 ± 923	4384 ± 839

Priming volumes			
Static circuit vol. (mL)	1406 ± 125	1412 ± 128	1386 ± 112

Medical history	Yes	No	Unknown
CHF	32 (5.6%)	535 (92.7%)	10 (1.7%)
CLD	6 (1.0%)	560 (97.1%)	11 (1.9%)
Smoking	230 (39.9%)	332 (57.5%)	15 (2.6%)
Diabetes	170 (29.5%)	396 (68.6%)	11 (1.9%)
Arrhythmia	37 (6.4%)	530 (91.9%)	10 (1.7%)
Hypertension	389 (67.4%)	181 (31.4%)	7 (1.2%)
Hyperlipidemia	260 (45.1%)	307 (53.2%)	10 (1.7%)
Non-cardiac VD	11 (1.9%)	553 (95.8%)	13 (2.3%)
Cerebral VD	16 (2.8%)	549 (95.1%)	12 (2.1%)
Renal failure	107 (18.6%)	463 (80.2%)	7 (1.2%)
Dialysis	52 (9.0%)	518 (89.8%)	7 (1.2%)

The data, except for laboratory data, are presented as mean ± S.D. or number (%). Laboratory data are presented as medians [IQR]. LV: left ventricular; CHF: chronic heart failure; CLD: chronic lung disease; VD: vascular disease; Hb: hemoglobin; TBV: total blood volume; CPB: cardiopulmonary bypass.

**Figure 2:**
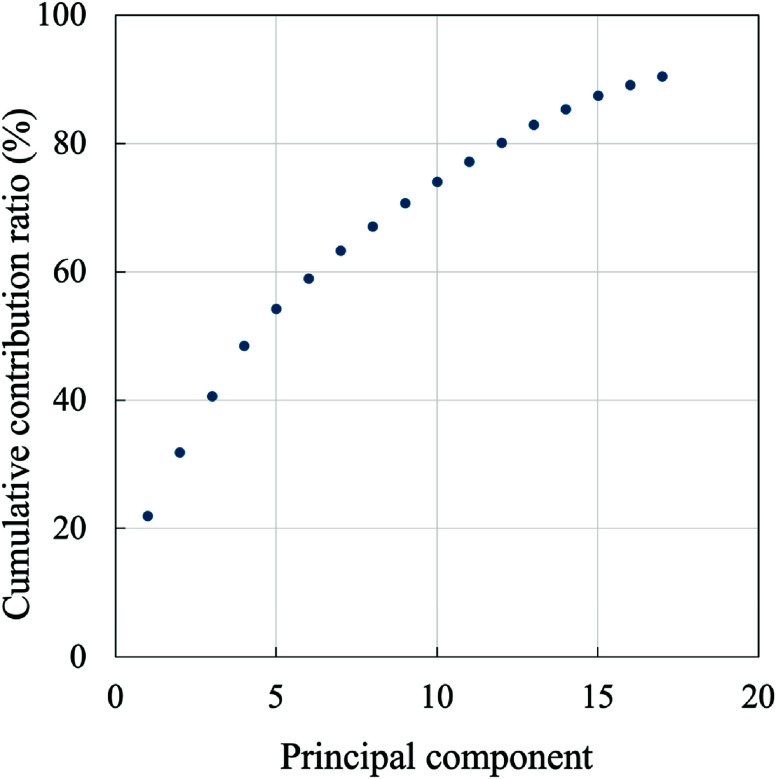
Cumulative explained variance. Principal components (PCs) are added sequentially from PC1 to PC17 (left to right); 90% cumulative explained variance is achieved with the first 17 PCs.

**Figure 3:**
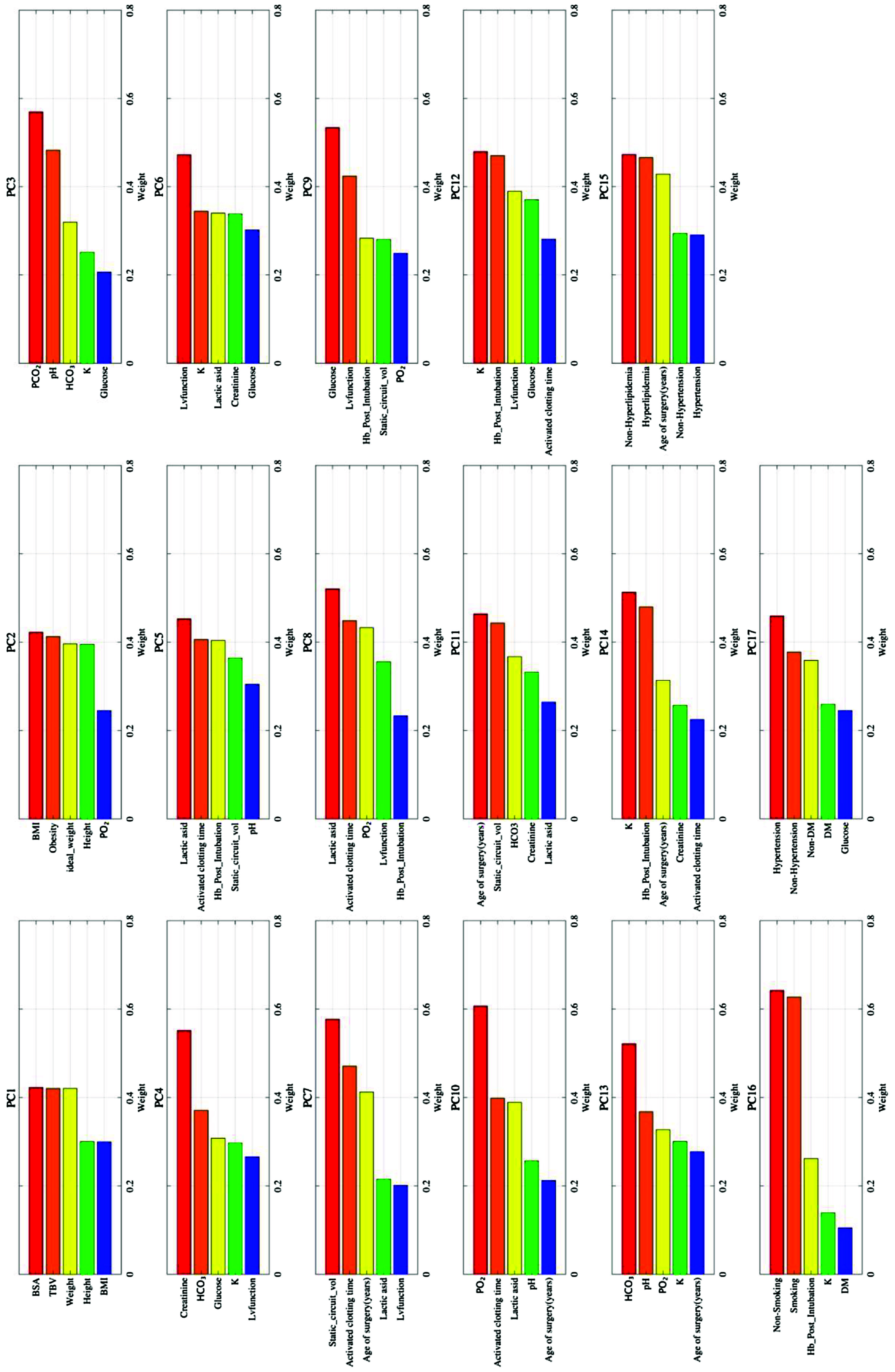
Weight of principal component analysis.

Figure 2 shows the scree plot of the principal components derived from PCA of the predictor matrix **X**∈R^*N*×*P*^, which comprises z-score standardized continuous variables and one-hot encoded categorical variables. From this plot, it was confirmed that 17 components were required to reach a cumulative explained variance of 90%. Figure 3 illustrates the five variables with the highest absolute loadings for each principal component. These results indicate that body weight was captured primarily by PC1, priming volume (static circuit volume) by PCs 5, 7, 9, and 11, and preoperative hemoglobin concentration by PCs 5, 8, 9, 12, 14, and 16. Table 2 presents the results of the generalized linear model fitted to the PCA-transformed predictors. The analysis revealed that intraoperative Hb_CPB_ was primarily determined by six principal components: PC1, PC2, PC5, PC8, PC12, and PC14.

**Table 2:** Regression results of PCA components for predicting Hb_CPB_.

Component	Estimate	*p*-value
Intercept	8.48	*<* 1 × 10^−308^
PC1	0.173	3*.*56 × 10^−29^
PC2	−0*.*0665	2.15 × 10^−3^
PC3	0.0207	3.70 × 10^−1^
PC4	−0*.*0329	1.76 × 10^−1^
PC5	0.245	4.79 × 10^−17^
PC6	0.0588	6.19 × 10^−2^
PC7	−0.0612	6.16 × 10^−2^
PC8	−0*.*1579	7.66 × 10^−6^
PC9	−0*.*0149	6.76 × 10^−1^
PC10	−0*.*0466	2.11 × 10^−1^
PC11	−0*.*0510	1.86 × 10^−1^
PC12	0.251	4.86 × 10^−10^
PC13	−0.0396	3.29 × 10^−1^
PC14	−0.2184	8.88 × 10^−7^
PC15	0.0533	2.55 × 10^−1^
PC16	0.0738	1.57 × 10^−1^
PC17	0.0822	1.63 × 10^−1^

**Figure 4:**
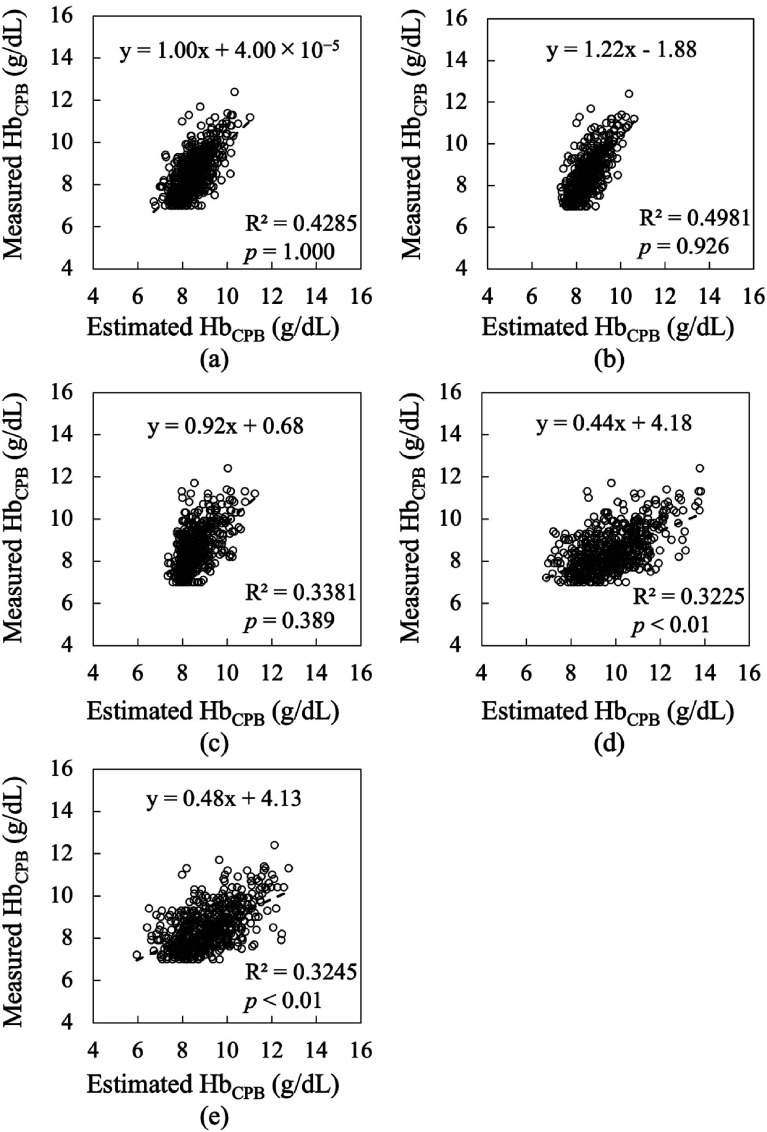
Correlation between measured Hb_CPB_ and estimated Hb_CPB_: (a) Hb_CPB_ was estimated based on GLM, (b) that based on SVR, (c) that based on MLP, (d) that calculated based on reference [11], and (e) that based on equation (6).

Figure 4 shows the comparison between measured Hb_CPB_ and predicted values based on pre-CPB variables, while Table 3 summarizes model performance indices. For each scatter plot in Figure 4, the ordinary least-squares regression slopes and *y*-intercepts – where better estimation is reflected by a slope close to 1 and a *y*-intercept close to 0 – are displayed. The slopes and intercepts (panels a–e) are as follows: (a) slope = 1.00, intercept = 4.00 × 10^−5^; (b) slope = 1.22, intercept = −1.88; (c) slope = 0.92, intercept = 0.68; (d) slope = 0.44, intercept = 4.18; (e) slope = 0.48, intercept = 4.13. A GLM fitted to the standardized predictor matrix **X**∈R^*N*×*P*^ achieved a coefficient of determination of $R_{\mathrm{GLM}}^{2}=\;0.429$, adjusted $R_{\mathrm{GLM}}^{2}=0.369$, and MSE_GLM_ = 0.797. However, multicollinearity in the input matrix was confirmed, resulting in a lack of full rank. Applying the same GLM to PCA-transformed predictors $X_{\mathrm{PCA}}\in R^{N\times P_{\mathrm{PCA}}}$ yielded $R_{\mathrm{PCR}}^{2}\;=\;0.371$ (adjusted $R_{\mathrm{PCR}}^{2}=\;0.352$) and MSE_PCR_ = 0.808. SVR on **X** with optimized hyperparameters *C* = 2.279, *γ* = 16.81, and *ε* = 0.594 yielded $R_{\mathrm{SVR}}^{2}\;=\;0.498$ and 10-fold CV MSE_SVR_ = 0.517, outperforming the GLM. When SVR was applied to **X**_PCA_ with *C* = 1.662, *γ* = 14.28, and *ε* = 0.0084, performance decreased to $R_{{\mathrm{SVR}}_{\mathrm{PCA}}}^{2}$ = 0.39 and CV ${\mathrm{MSE}}_{{\mathrm{SVR}}_{\mathrm{PCA}}}=0.52$. The MLP model, tuned via nested 5-fold CV, selected one hidden-layer architecture of 4 neurons, with Resilient Backpropagation, a learning rate of 10^−2^, and early stopping tolerance of 10. On **X**, this configuration yielded $R_{\mathrm{MLP}}^{2}$ = 0.332 ± 0.033 and MSE_MLP_ = 0.669 ± 0.086. When applied to **X**_PCA_, the MLP achieved *R*^2^ = 0.352 ± 0.054 and MSE = 0.646 ± 0.068. The coefficients of determination from both MLP configurations were lower than those from SVR. Previously published Hb_CPB_ prediction formulas yielded *R*^2^ = 0.3225, MSE = 2.98 based on [19], and *R*^2^ = 0.3245, MSE = 1.48 based on [18], both substantially lower than SVR performance.

**Table 3:** Comparison of prediction performance of hemoglobin prediction models.

Model	MSE	*R*^2^ (adjusted *R*^2^)
GLM	0.797	0.429 (0.369)
PCR	0.808	0.371 (0.352)
SVR	0.517	0.489
SVR with PCA	0.520	0.390
MLP	0.669 ± 0.086	0.332 ± 0.033
MLP with PCA	0.646 ± 0.068	0.352 ± 0.054

MSE: mean squared error; *R*^2^: coefficient of determination; GLM: general linear model; PCR: principal component regression; SVR: support vector regression; PCR: principal component analysis; MLP: multilayer perceptron.

**Figure 5:**
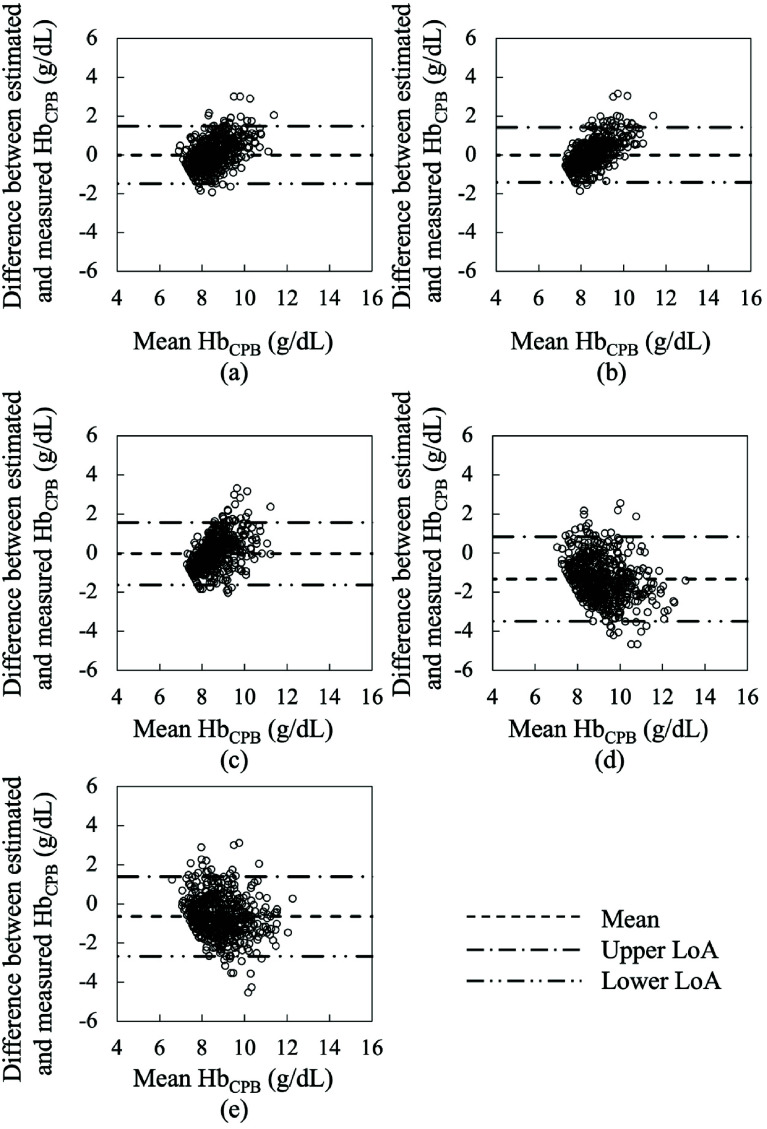
Bland–Altman analysis between measured Hb_CPB_ and estimated Hb_CPB_: (a) estimated Hb_CPB_ was estimated based on GLM, (b) that based on SVR, (c) that based on MLP, (d) that calculated based on reference [11], and (e) that based on equation (6).

Figure 5 displays the Bland–Altman analysis results. The bias between predicted and measured GLM values was –5 × 10^−7^ g/dL (95% CI: −0.0654 to 0.0654 g/dL), with 95% LoA of [–1.57 g/dL, 1.57 g/dL]. For SVR, the bias was –0.0028 g/dL (95% CI: −0.0619 to 0.0562 g/dL) with LoA of [–1.41 g/dL, 1.41 g/dL]. For MLP, the bias was –0.029 g/dL (95% CI: −0.0963 to 0.0375 g/dL), and LoA was [–1.63 g/dL, 1.57 g/dL]. In contrast, the conventional methods had a bias of –1.33 g/dL (95% CI: –1.41 to –1.24 g/dL) and LoA of [–3.49 g/dL, 0.831 g/dL] for the reference [11] and a bias of –0.64 g/dL (95% CI: –0.72 to –0.555 g/dL) and LoA of [–2.67 g/dL, 1.39 g/dL] for the equation (5), respectively. The 95% CI for bias did not include zero, indicating systematic error. In comparison, CIs from GLM, SVR, and MLP included zero, suggesting no statistically significant bias and the absence of systematic error. Biases between measured and predicted Hb_CPB_ from GLM, SVR, and MLP were not statistically significant (GLM: *p* = 1.000, *d* = −6.5 × 10^−7^; SVR: *p* = 0.926, *d* = −0.004; MLP: *p* = 0.389, *d* = −0.036). Conversely, biases from conventional models were confirmed (reference [11]: *p* < 0.01, *d* = −1.21; equation (5): *p* < 0.01, *d* = −0.617).

## Discussion

The GLM demonstrated moderate goodness-of-it despite the multicollinearity present in the input parameter matrix **X**∈R^*N*×*P*^. In contrast, implementing PCR resulted in reduced model performance across all evaluation metrics compared with the GLM. In PCR, dimensionality reduction is driven solely by the variance structure of the predictor matrix; therefore, principal components that explain substantial variance but have limited correlation with the response variable may still be retained as leading components. In this study, the first 17 principal components were adopted, collectively accounting for 90% cumulative explained variance in the predictor matrix. While this approach preserved most of the predictor information, it may have failed to isolate features most relevant to ${\mathrm{Hb}}_{\mathrm{CPB}}$. Furthermore, the first principal component accounted for more than 20% of the total variance, and PC1 and PC2 were primarily composed of weight, height, TBV, BSA, BMI, ideal weight, and obesity measures, supporting the interpretation that TBV-related factors remain central to Hb_CPB_ estimation. As a result, the PCR model may not have adequately captured the most predictive features of Hb_CPB_, likely contributing to its reduced performance, as reflected in the lower coefficient of determination and higher MSE compared with the GLM using the full predictor set. Traditional predictors used to estimate Hb_CPB_ – including body weight, priming volume (static circuit volume), and preoperative Hb concentration – were embedded within the principal components retained by PCR and were selected as input features for both the SVR and MLP models. Nevertheless, several of the principal components used in Hb_CPB_ prediction did not primarily reflect these conventional parameters. Therefore, Hb_CPB_ cannot be fully explained by body weight, priming volume, and preoperative Hb concentration alone, as suggested by conventional approaches. At the same time, while models using PCs 1–17 showed better performance than the conventional formula, we cannot presently ascribe the improvement to specific components without dedicated ablation or feature-contribution analyses. These findings highlight the influence of additional confounding variables, including age and comorbidities.

Although model development and training require computational resources (workstation- or cloud-based), inference is lightweight and can be executed on common devices (e.g., a standard laptop or smartphone) with low latency and no specialized hardware. This enables point-of-care use in the operating room as a simple calculator or as a module integrated into existing perfusion workflows. Accordingly, the trained model can be delivered either (i) within the EHR as a point-of-care calculator or clinical decision support widget that auto-populates inputs from routinely collected pre-CPB data, or (ii) on perfusion hardware (e.g., as a vendor plug-in) to present predictions alongside pump parameters. After deployment, the model can operate offline, with input variables populated automatically from routine pre-CPB data, minimizing manual entry and cognitive load.

Among the nonlinear models, SVR demonstrated a higher coefficient of determination and lower MSE compared with both the GLM and PCR, indicating that SVR had superior predictive power. In contrast, applying PCA prior to SVR led to a decreased coefficient of determination, likely due to the exclusion of ${\mathrm{Hb}}_{\mathrm{CPB}}$-related features during the PCA process, as these were among the bottom 10% of principal components that were subsequently discarded. The MLP model, incorporating Levenberg–Marquardt–based parameter optimization and early stopping, achieved stable training. When applied to the standardized predictor matrix, MLP showed slightly lower performance than SVR but still achieved a moderate goodness-of-fit. Overall, SVR and MLP, which perform nonlinear function estimation and embed variable selection within the model, outperformed linear models, including conventional approaches, in predicting outcomes from high-dimensional medical data.

Although the best-performing method yielded a modest *R*^2^, agreement relevant to bedside use is better captured by Bland–Altman analysis [31, 32]: the new methods demonstrated minimal bias and narrower limits of agreement than the conventional formulas (Figure 5), indicating improved agreement despite a moderate *R*^2^ compared with previous methods. Finally, while models using multiple principal components outperformed the conventional formula, we cannot presently ascribe the improvement to specific components without dedicated ablation or feature-contribution analyses; this is noted as a limitation and will be addressed in future work.

Model development and training require computational resources; these activities can be conducted cost-effectively on a workstation or in the cloud using open-source machine-learning frameworks. After training, inference is lightweight, runs on commodity devices without accelerators, and can be embedded in existing EHR or perfusion-system workflows with minimal latency. From a clinical perspective, more accurate prediction of Hb_CPB_ at CPB initiation supports optimized transfusion strategies – reducing the likelihood of both under- and over-transfusion – and may help lower hemodilution-related risks while standardizing practice across teams. We therefore view the cost–benefit balance as favorable, while acknowledging that prospective evaluation of clinical outcomes and a formal cost-effectiveness analysis are warranted in future work.

## Limitations

While the proposed models using PCs 1–17 outperformed the conventional formula, we cannot, at present, attribute this improvement to specific principal components without dedicated ablation or feature-contribution analyses. We acknowledge this as a limitation and plan to investigate the contribution of individual components and underlying variables in future work.

Although inference is feasible on standard devices, clinical integration remains ongoing. Both EHR-based and device-based (perfusion console) deployments are feasible, but practical adoption will require: (A) interoperability with hospital systems and/or perfusion consoles, (B) a user interface suited to intraoperative workflow, (C) prospective validation with monitoring for model drift, and (D) security and access control consistent with institutional policies. We plan to evaluate these aspects and conduct prospective usability and safety assessments prior to routine operating-room adoption.

## Data Availability

Data available upon request from the corresponding author.

## Supplementary material

S1. Generalized Linear Model (GLM). S2. Support Vector Regression (SVR). S3. Multilayer Perceptron (MLP). S4. Dimensionality Reduction via PCA. S5. Cross-validation, Metrics, and Comparative Baselines. The supplementary material of this article is available at https://ject.edpsciences.org/10.1051/ject/2025071/olm.
