# Supplementary material for: Estimation of hemoglobin concentration at the initiation of cardiopulmonary bypass using support vector regression
Source: J Extra Corpor Technol. 2026 Jun 19;58(2):128–38. doi: 10.1051/ject/2025071 (PMC13281317; doi:10.1051/ject/2025071)
Supplement: Supplementary file 1 — S1. Generalized Linear Model (GLM). S2. Support Vector Regression (SVR). S3. Multilayer Perceptron (MLP). S4. Dimensionality Reduction via PCA. S5. Cross-validation, Metrics, and Comparative Baselines. [file ject-58-128-s1.pdf]

Supplemental material

S1. Generalized Linear Model (GLM)

For standardized predictors $X \in \mathbb{R}^{N\times P}$ and target $y \in \mathbb{R}^{N}(\mathrm{Hb_{CPB}})$, we fit a Gaussian-identity GLM:

$$y = \beta_0 + X\beta + \varepsilon, \quad \varepsilon \sim \mathcal{N}(0, \sigma^2 I). \qquad \text{(s-i)}$$

Parameters (β_0, β) were estimated by maximum likelihood (equivalently, least squares under (i)). Implementation used MATLAB fitglm (MathWorks) with default options.

S2. Support Vector Regression (SVR)

We used ε-SVR with radial basis function (RBF) kernel as shown in the following equation:

$$K(x_i, x_j) = \exp\left(-\frac{\| x_i - x_j\|^2}{2\sigma^2}\right). \qquad \text{(s-ii)}$$

A ε-SVR model is obtained by solving the convex optimization problem as follows:

$$\min_{w,b,\xi,\xi^*} \frac{1}{2}\|w\|^2 + C\sum_{i=1}^{n} \xi_i + \xi_i^*, \qquad \text{(s-iii)}$$

$$\text{subject to} \begin{cases} y_i - (w^T\phi(x_i) + b) \le \varepsilon + \xi_i, \\ (w^T\phi(x_i) + b) - y_i \le \varepsilon + \xi_i^*, \\ \xi_i, \xi_i^* \ge 0, \quad i = 1, \dots, n, \end{cases}$$

where ϕ is the mapping to the feature space, C is the penalty constant (BoxConstraint), and ε is the width of the epsilon-insensitive zone. The predicted Hb concentration $\hat{y}(x)$ is then expressed as a linear combination of support vectors, as follows:

$$\hat{y}(x) = \sum_{i \in \boldsymbol{S}} \alpha_i, K(x_i, x) + b\,, \qquad \boldsymbol{S} = \{i | \alpha_i \neq 0\}. \tag{s-iv}$$

We tuned (C, σ, γ, ε) by cross-validation. Implementation used MATLAB fitrsvm with RBF kernel; expected-improvement-plus criterion in 10-fold CV guided selection.

S3. Multilayer Perceptron (MLP)

We used a feed-forward network with $L-1$ hidden layers. For an input $x \in \mathrm{R}^{\mathrm{P}}$, the layer outputs were defined as:

$$\mathbf{h}^{(l)} = \phi\left(\mathbf{W}^{(l)}\mathbf{h}^{(l-1)} + \mathbf{b}^{(l)}\right), \qquad \mathbf{h}^{(0)} = \mathbf{X}. \tag{s-v}$$

with the final prediction given by:

$$\hat{\boldsymbol{y}} = \mathbf{W}^{(L)}\mathbf{h}^{(L-1)} + \mathbf{b}^{(L)}, \tag{s-vi}$$

The weights $\{W^{(\mathrm{l})}, b^{(l)}\}$were learned by backpropagation using either the Levenberg–Marquardt or Resilient Backpropagation algorithm [30, 31], with learning rates 10^{-3} or 10^{-4} and early-stopping patience of 10 or 20 epochs. Model selection followed a nested cross-validation scheme with 5 outer folds and a 5-fold inner grid search over hidden-layer sizes [4], [8], [16],

[32], [32, 16], [64, 32], [128, 64], algorithm $\in\{\text{LM},\text{RProp}\}$, learning rate $\in\{10^{-3}, 10^{-4}\}$, and early-stopping tolerance $\in\{10,20\}$. On the full standardized predictor matrix X, the selected configuration was [4] (one hidden layer with 4 neurons) trained with LM at a learning rate of 10^{-3}, early stopping of 10 epochs, and L2 weight decay of 0.1 [32]; five random initializations were run and the model with the lowest validation MSE was retained.

S4. Dimensionality Reduction via PCA.

Continuous variables were *z*-score standardized and categorical variables were one-hot encoded. Principal component analysis (PCA) was then applied to the full predictor matrix to obtain the score matrix $P \in \mathbb{R}^{N\times p_{\text{PCA}}}$. The retention rule was prespecified as “keep the smallest number of components whose cumulative explained variance reaches at least 90%.” Component loadings were computed to support interpretability.

S5. Cross-validation, Metrics, and Comparative Baselines.

For model validation, SVR/MLP used standard train–validation splits with reporting on the final fit; SVR employed 10-fold cross-validation for hyperparameter selection and cross-validated

error estimates; and MLP used nested 5 × 5 cross-validation for model selection with outer-fold performance reported. Model performance was summarized uniformly using R^2 and MSE, and agreement between predicted and measured values was assessed with Bland–Altman analysis (bias and 95% limits of agreement). Two published TBV-based formulas were included as comparative baselines; all models (baselines and proposed) were evaluated under the same preprocessing and validation procedures.
